# Supplementary figures and images for: Prediction of cancer cell sensitivity to natural products based on genomic and chemical properties
Source: PeerJ. 2015 Nov 26;3:e1425. doi: 10.7717/peerj.1425 (PMC4671159; doi:10.7717/peerj.1425)

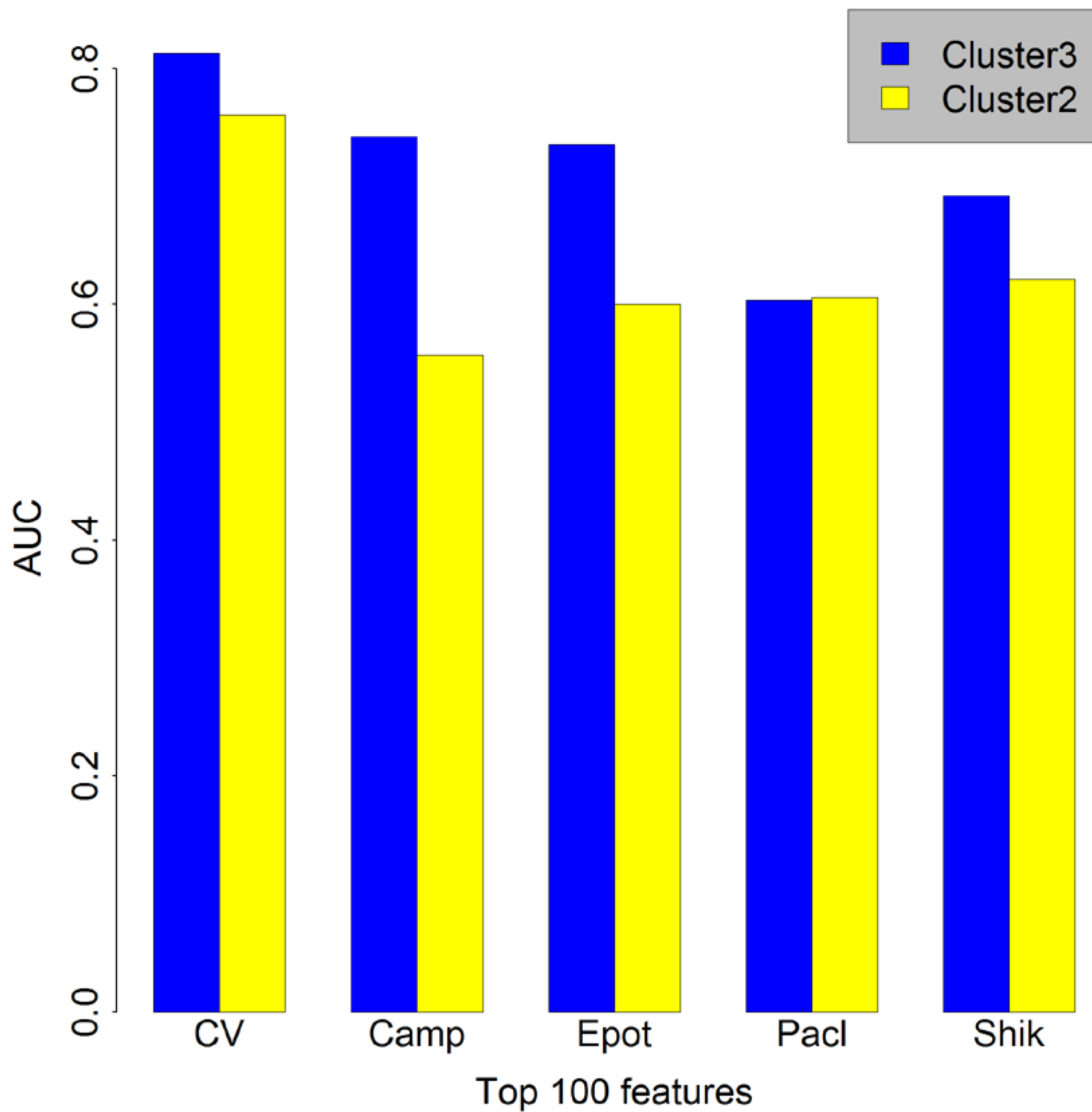

Supplement: Figure S1 — Bar chart showing in the case K = 3 (blue) we obtained a higher AUC than in the case K = 2 when features number is set as 100. Cluster3, the case K = 3; Cluster2, the case K = 2; CV, cross validation; Camp, Camptothecin; Epot, Epothilone B; Pacl, Paclitaxel; Shik, Shikonin; AUC, Area under the curve. [file peerj-03-1425-s001.pdf]

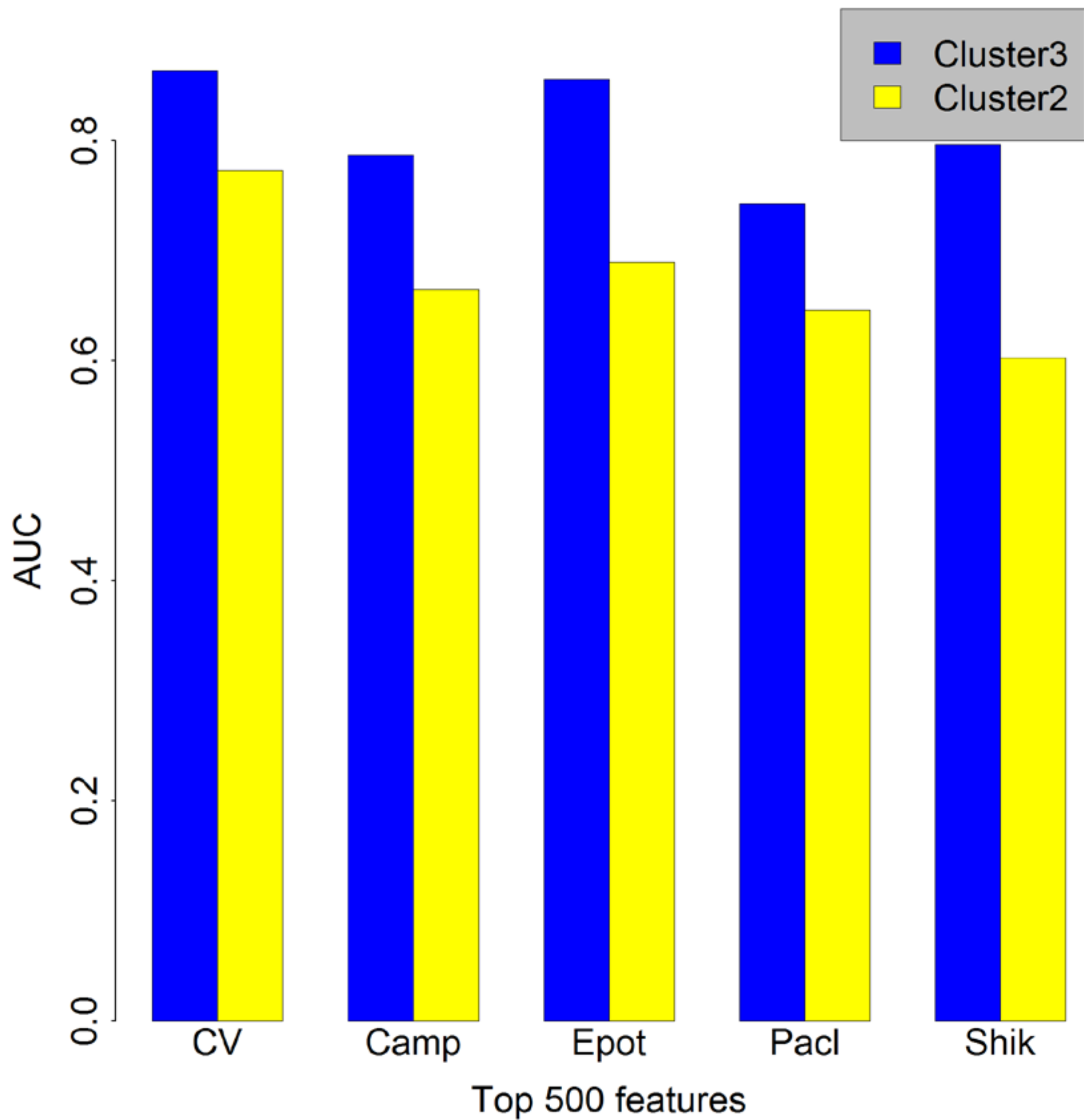

Supplement: Figure S2 — Bar chart showing in the case K = 3 (blue) we obtained a higher AUC than in the case K = 2 when features number is set as 500. Cluster3, the case K = 3; Cluster2, the case K = 2; CV, cross validation; Camp, Camptothecin; Epot, Epothilone B; Pacl, Paclitaxel; Shik, Shikonin; AUC, Area under the curve. [file peerj-03-1425-s002.pdf]
